# In Vitro Toxicological Profile of Labetalol-Folic Acid/Folate Co-Administration in H9c2(2-1) and HepaRG Cells

**DOI:** 10.3390/medicina58060784

**Published:** 2022-06-10

**Authors:** Robert Rednic, Iasmina Marcovici, Razvan Dragoi, Iulia Pinzaru, Cristina Adriana Dehelean, Mirela Tomescu, Diana Aurora Arnautu, Marius Craina, Adrian Gluhovschi, Mihaela Valcovici, Aniko Manea

**Affiliations:** 1 Faculty of Medicine, “Victor Babes” University of Medicine and Pharmacy Timisoara, Eftimie Murgu Square No.2, 300041 Timisoara, Romania; rednic.robert@umft.ro (R.R.); tomescu.mirela@umft.ro (M.T.); aurora.bordejevic@umft.ro (D.A.A.); craina.marius@umft.ro (M.C.); gluhovschi.adrian@umft.ro (A.G.); mvalcovici@cardiologie.ro (M.V.); aniko180798@yahoo.com (A.M.); 2 Faculty of Pharmacy, “Victor Babes” University of Medicine and Pharmacy Timisoara, Eftimie Murgu Square No.2, 300041 Timisoara, Romania; iasmina.marcovici@umft.ro (I.M.); cadehelean@umft.ro (C.A.D.); 3 Research Center for Pharmaco-Toxicological Evaluations, Faculty of Pharmacy, “Victor Babes” University of Medicine and Pharmacy Timisoara, Eftimie Murgu Square No.2, 300041 Timisoara, Romania

**Keywords:** labetalol, folic acid, folate, dietary supplements, cytotoxicity

## Abstract

*Background and Objectives:* The consumption of dietary supplements has increased over the last decades among pregnant women, becoming an efficient resource of micronutrients able to satisfy their nutritional needs during pregnancy. Furthermore, gestational drug administration might be necessary to treat several pregnancy complications such as hypertension. Folic acid (FA) and folate (FT) supplementation is highly recommended by clinicians during pregnancy, especially for preventing neural tube birth defects, while labetalol (LB) is a β-blocker commonly administered as a safe option for the treatment of pregnancy-related hypertension. Currently, the possible toxicity resulting from the co-administration of FA/FT and LB has not been fully evaluated. In light of these considerations, the current study was aimed at investigating the possible in vitro cardio- and hepato-toxicity of LB-FA and LB-FT associations. *Materials and Methods:* Five different concentrations of LB, FA, FT, and their combination were used in myoblasts and hepatocytes in order to assess cell viability, cell morphology, and wound regeneration. *Results:* The results indicate no significant alterations in terms of cell viability and morphology in myoblasts (H9c2(2-1)) and hepatocytes (HepaRG) following a 72-h treatment, apart from a decrease in the percentage of viable H9c2(2-1) cells (~67%) treated with LB 150 nM–FT 50 nM. Additionally, LB (50 and 150 nM)–FA (0.2 nM) exerted an efficient wound regenerating potential in H9c2(2-1) myoblasts (wound healing rates were >80%, compared to the control at 66%), while LB-FT (at all tested concentrations) induced no significant impairment to their migration. *Conclusions:* Overall, our findings indicate that LB-FA and LB-FT combinations lack cytotoxicity in vitro. Moreover, beneficial effects were noticed on H9c2(2-1) cell viability and migration from LB-FA/FT administration, which should be further explored.

## 1. Introduction

The last few decades have seen extensive growth in the consumption of dietary supplements (DTs), which can be defined as oral products administered with the purpose of correcting one’s dietary deficiencies. DTs containing micronutrients (e.g., vitamins and minerals) play a vital role in satisfying maternal nutritional requirements during pregnancy, which are insufficiently met through their daily diets [1]. Furthermore, micronutrient deficiency during pregnancy has been correlated with serious maternal and fetal health issues, such as congenital malformations and pre-eclampsia [1,2]. Thus, in order to prevent possible nutrient inadequacies during pregnancy, DTs are highly recommended by clinicians, becoming a common practice among pregnant women worldwide [1,3]. In particular, folic acid (FA) supplementation (at intake levels of 400 µg to 5 mg/day) is recommended for all women of reproductive age both in the periconceptional period and up until the 12th week of pregnancy [4,5].

FA is a synthetic dietary supplement belonging to a family of water-soluble vitamins typically referred to as “folates” or “vitamin B9” [6,7]. FA plays an essential role in DNA synthesis, repair, and methylation [8], and its maternal supplementation has been correlated with a reduced risk of developing neural tube birth defects [5]. However, FA needs to undergo several transformations within the human body in order to become metabolically active [9]. This process includes the reduction of FA to dihydrofolate (DHF) and tetrahydrofolate (THF), followed by its conversion to the biologically active 5-methyltetrahydrofolate (5-MTHF) [6]. 5-MTHF represents the predominant form found in plasma (>90% of total folate) and the main active metabolite of the ingested FA [9].

Many physiological changes occur during pregnancy to enable proper placental and fetal development. Unfortunately, these changes might affect preexisting maternal diseases or even result in pregnancy-related disorders [10]. Hypertension represents the most commonly encountered medical complication during pregnancy (up to 10% of pregnancies) and is the leading cause of maternal, fetal, and neonatal morbidity and mortality worldwide [11,12,13]. Pregnancy-related hypertensive disorders, which have been associated with an increased risk of developing maternal type 2 diabetes and cardiovascular disease in later life [14,15], cover a broad spectrum of conditions, including chronic hypertension, gestational hypertension, pre-eclampsia, and pre-eclampsia superimposed on chronic hypertension [16,17]. While the definitive treatment for acute hypertensive syndromes occurring during pregnancy is delivery, antihypertensive medication is one of the most employed management strategies in preventing maternal cerebrovascular and cardiac complications. Antihypertensive agents that are widely recommended to control maternal hypertension during pregnancy should not impair the uteroplacental and fetal circulation, and present limited toxicity to the fetus [16]. In current practice, the first-line pharmacological treatment of pregnancy-related hypertensive disorders is based on antihypertensive drugs, such as methyldopa and labetalol, while the second-line strategy includes nifedipine, verapamil, clonidine, and hydrochlorothiazide [18].

Labetalol (LB) is a β-blocker medication commonly recommended as a safe option for the treatment of maternal hypertension during pregnancy [19,20]. Compared to other β-blockers, LB contains both selective α-adrenergic and non-selective β-adrenergic blocking activities in a single agent and preserves the uteroplacental blood flow. However, despite its favorable safety profile, LB has been associated with several side effects, including hypotension, bradycardia, cardiac impairment, and maternal hepatotoxicity [21,22].

The leading hypothesis of this study was that labetalol-folic acid or folate co-administration during pregnancy might result in deleterious side effects, which, to the best of our knowledge, lack any investigation so far. Thereafter, the aim of the current paper was to portray an in vitro toxicological profile of labetalol associated with folic acid and folate using healthy myoblasts and hepatocytes as models for compound-induced cardio- and hepato-toxicity.

## 2. Materials and Methods

### 2.1. Reagents

Labetalol hydrochloride (LB), folic acid, folate (as 5-Methyltetrahydrofolic acid), trypsin-EDTA solution, phosphate saline buffer (PBS), dimethyl sulfoxide (DMSO), fetal calf serum (FCS), penicillin/streptomycin, insulin from bovine pancreas, hydrocortisone 21-hemisuccinate sodium salt, and MTT (3-(4,5 dimethylthiazol2-yl)-2,5-diphenyltetrazolium bromide) reagent were purchased from Sigma Aldrich, Merck KgaA (Darmstadt, Germany). Dulbecco’s Modified Eagle Medium (DMEM; ATCC^®^ 30-2002™) was purchased from ATCC (American Type Cell Collection, Lomianki, Poland), and William’s E Medium was purchased from Gibco Waltham, MA, USA.

### 2.2. Cell Culture

Myoblast (heart, myocardium) immortalized cell line (H9C2(2-1); code CRL-1446™) was provided as a frozen vial by ATCC. The cells were cultured in their specific media (DMEM). Hepatic immortalized cell line (HepaRG; code HPRGC10) was purchased from ThermoFisher Scientific (Gibco Waltham, MA, USA) and cultured in William’s E Medium enriched with insulin and hydrocortisone 21-hemisuccinate sodium salt at final concentrations of 4 µg/mL and 50 µM, respectively. Both media contained a 10% FCS and 1% penicillin (100 U/mL)/streptomycin (100 µg/mL) mixture. The cells were kept in an incubator at 37 °C and 5% CO_2_ during the experiments.

### 2.3. Cellular Viability

To evaluate the impact of LB, FA, and FT on the viability of myoblasts and hepatocytes, the MTT technique was applied. Briefly, H9c2(2-1) and HepaRG cells were cultured in 96-well plates (10^4^ cells/well) and stimulated for 72 h with LB (10, 25, 50, 100, and 150 nM), FA, and FT (0.2, 1, 10, 25, and 50 nM). The stock solution of LB was prepared by dissolving the substance in distilled water, while the FA and FT stock solutions were prepared in DMSO. The FA and FT solutions were further diluted in distilled water and culture media for in vitro testing. At the end of the treatment, fresh media (100 µL) and MTT reagent (10 µL) were added to the wells, and the plates were incubated at 37 °C for another 3 h. Finally, the solubilization solution (100 µL/well) was added to each well. The plates were kept at room temperature for 30 min, protected from light, and the absorbance values were measured at two wavelengths (570 and 630 nm) using Cytation 5 (BioTek Instruments Inc., Winooski, VT, USA).

### 2.4. Cellular Morphology and Confluence

To verify the influence of LB-FA and LB-FT associations on the morphology and confluence of H9c2(2-1) and HepaRG cells, a bright field microscopic examination was performed. The cells were photographed at the end of the 72-h treatment period using Cytation 1 (BioTek Instruments Inc., Winooski, VT, USA). The obtained pictures were processed using the Gen5™ Microplate Data Collection and Analysis Software (BioTek Instruments Inc., Winooski, VT, USA).

### 2.5. Wound Regeneration

The regenerating potential of LB, FA, FT, and their combinations on healthy H9c2(2-1) and HepaRG cells following wounding was evaluated by applying the wound healing (scratch) assay. In brief, the cells (10^5^ cells/mL/well) were cultured in 12-well plates, and a manual scratch was made in the middle of each well. The cells were treated with the test compounds for 24 h, and representative images were taken at 0 h and 24 h using an Olympus IX73 inverted microscope equipped with a DP74 camera. The wound widths were measured at the end of the treatment with CellSense Dimension 1.17 (Olympus, Tokyo, Japan). The quantification of the effects in terms of cell migration was performed by calculating the wound healing rates (%) according to a formula used in our previous work [23,24].

### 2.6. Statistical Analysis

All data are expressed as the means ± SD, and the differences were compared by one-way ANOVA analysis followed by Dunnett’s multiple comparisons post-test. The used software was GraphPad Prism version 9.2.0 for Windows (GraphPad Software, San Diego, CA, USA, www.graphpad.com). The statistically significant differences among the data are marked with * (* *p* < 0.05; ** *p* < 0.01; *** *p* < 0.001; **** *p* < 0.0001).

## 3. Results

### 3.1. Cellular Viability

**Figure 1:**
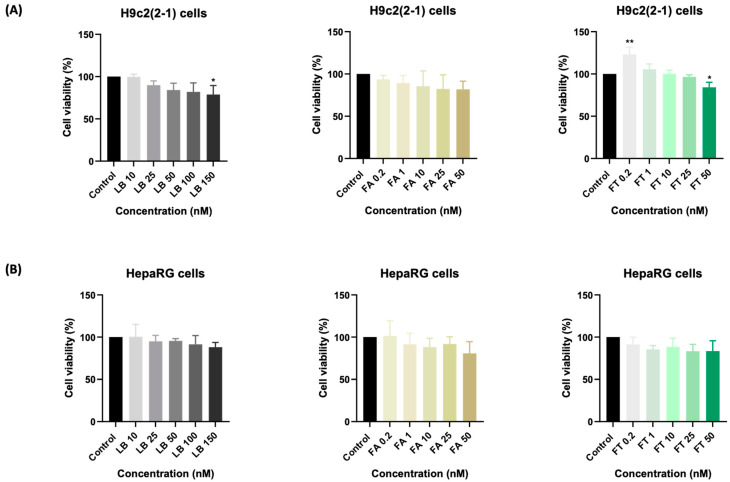
The impact of a 72-h treatment with labetalol (LB), folic acid (FA), and folate (FT) on the viability of (**A**) H9c2(2-1) and (**B**) HepaRG cells. The data are expressed as the mean values ± SD of three independent experiments performed in triplicate (*n* = 3). The statistical differences between the control and the treated group were quantified by one-way ANOVA analysis followed by Dunnett’s multiple comparisons post-test (* *p* < 0.05; ** *p* < 0.01).

The effects of LB, FA, FT, and their combinations on the viability of healthy myoblasts were evaluated following a prolonged treatment (72 h). LB induced a dose-dependent decrease in the viability of H9c2(2-1) cells, the most prominent effect being recorded at 150 nM (~79%) (Figure 1A). Similarly, FA lowered the cell viability in a concentration-dependent manner up to ~82% at 50 nM (Figure 1B). FT determined a significant increase in the percentage of viable cells at the lowest concentration of 0.2 nM (~127%), while at the highest concentration of 50 nM, the viability was reduced to ~83%. A similar trend was noticed in the HepaRG cells (Figure 1B), their viability being dose-dependently reduced following the single treatments. The lowest cell viability percentages were registered at the highest concentrations tested, as follows: LB 150 nM—88%, FA 50 nM—81%, and FT 50 nM—84%. 

**Figure 2:**
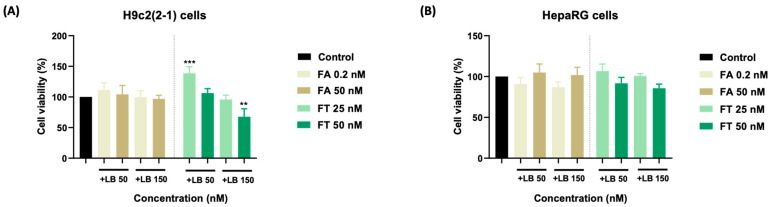
The impact of a 72-h treatment with LB–FA and LB–FT on the viability of (**A**) H9c2(2-1) cells and (**B**) HepaRG cells. The data are expressed as the mean values ± SD of three independent experiments performed in triplicate (*n* = 3). The statistical differences between the control and the treated group were quantified by one-way ANOVA analysis followed by Dunnett’s multiple comparisons post-test (** *p* < 0.01; *** *p* < 0.001).

The impact of the LB–FT combination on the myoblasts’ viability was concentration-dependent. The addition of LB 50 nM to the FT treatment exerted a stimulatory effect (138% for FT 25 nM, and 106% for FT 50 nM). On the other hand, LB 150 nM combined with FT 25 and 50 nM reduced the cell viability to ~96% and ~68%, respectively. The impact of LB combined with FA on the viability of H9c2(2-1) cells was insignificant (compared to the control) (Figure 2A). In the case of the HepaRG cells (Figure 2B), a reduction in cell viability was observed following the combination of LB 50 and 150 with FA 0.2 nM (~91%, and ~87%, respectively) and FT 50 nM (~92% and ~86%, respectively). The other treatment regimens led to cell viabilities that were similar to untreated cells (around the value of 100%).

### 3.2. Cellular Morphology and Confluence

**Figure 3:**
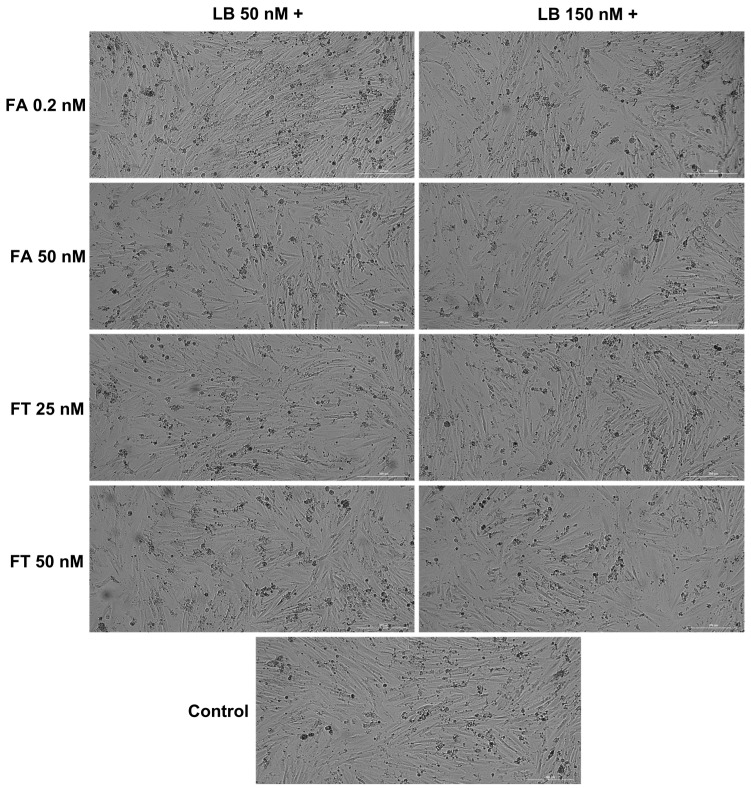
Morphological analysis of H9c2(2-1) myoblasts following the 72-h combined treatment of LB 50 and 150 nM with FA (0.2 and 50 nM) and FT (25 and 50 nM).

**Figure 4:**
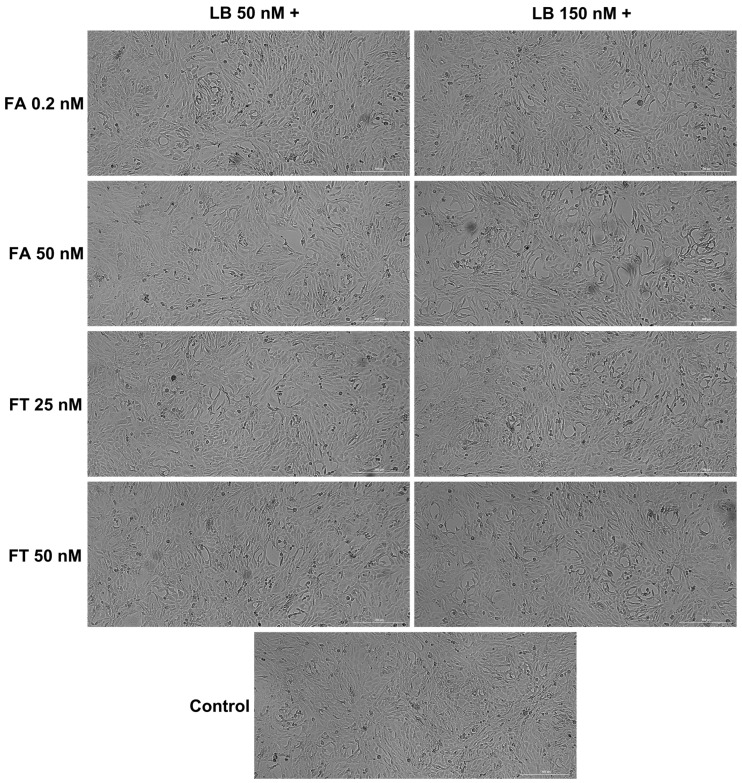
Morphological analysis of HepaRG hepatocytes following the 72-h combined treatment of LB 50 and 150 nM with FA (0.2 and 50 nM) and FT (25 and 50 nM).

The morphological evaluation (Figure 3 and Figure 4) indicated no significant confluence changes in the H9c2(2-1) and HepaRG cells following the 72 h treatment with LB–FA and LB–FT. However, the addition of LB 150 nM induced a slight decrease in the cell confluence (most visible for FT 50 nM in the H9c2(2-1) cells and FA 50 nM in the HepaRG cells).

### 3.3. Wound Regeneration

**Figure 5:**
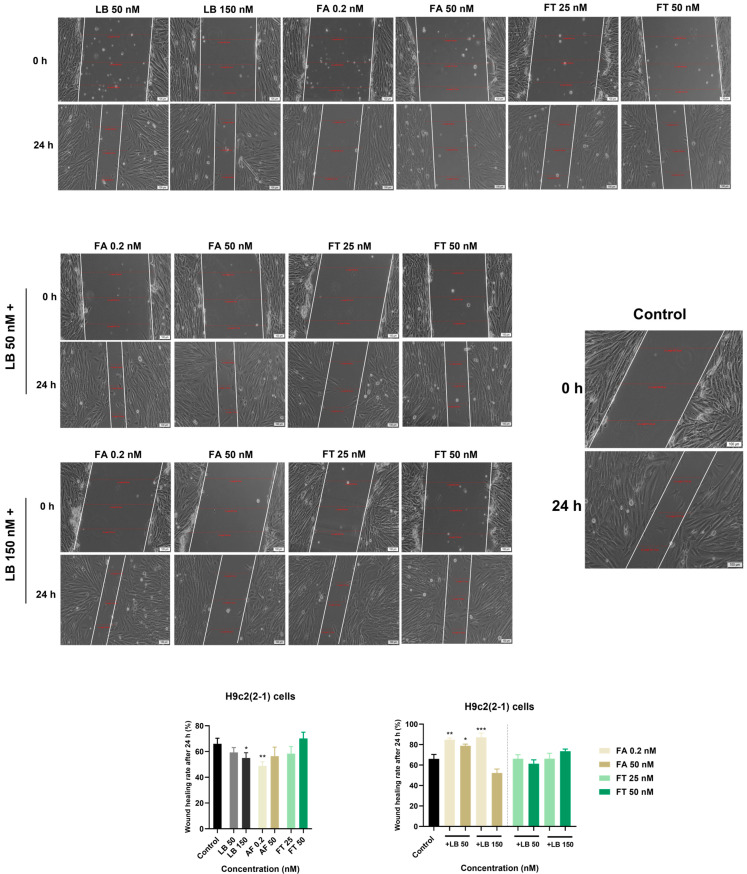
Representative images highlighting the impact of LB, FA, FT, and their combinations on wound regeneration in H9c2(2-1) cells following a 24 h treatment, and graphic representations of the calculated wound healing rates for each treatment regimen (single and combined). The white lines indicate the wound area. The scale bars indicate 100 µm. The data are expressed as the mean values ± SD of three independent experiments performed in triplicate. The statistical differences between the control and the treated group were quantified by one-way ANOVA analysis followed by Dunnett’s multiple comparisons post-test (* *p* < 0.05; ** *p* < 0.01; *** *p* < 0.001).

In order to assess whether LB, FA, FT, and their combinations interfere with the migration of H9c2(2-1) cells, a wound healing assay was performed. The wound healing rate of untreated H9c(2-1) cells (control) reached the value of ~66% (out of 100%, indicating full scratch closure) following 24 h. Compared to the control, the single treatment of the H9c2(2-1) cells with LB and FA inhibited wound healing at both tested concentrations following 24 h of treatment. FT, on the other hand, exerted an inhibitory effect at 25 nM, while slightly stimulating wound regeneration at 50 nM with a wound healing rate of ~70%. The results also suggest that all combinations, except for LB 150 nM–FA 50 nM (wound healing rate = ~53%), stimulate cell migration and wound healing following 24 h of treatment. Compared to untreated cells, the strongest stimulatory effects were recorded when LB (at concentrations of 50 and 150 nM) was combined with FA 0.2 nM with wound healing rates of ~85% and ~88%, respectively. The results are presented in Figure 5.

## 4. Discussion

The safety of the individual administration of folic acid/folate and labetalol during pregnancy has already been documented in the literature [5,25]; however, studies regarding the possible toxicity caused by their co-administration are scarce. Driven by the lack of data on this subject, the current study was designed to examine the potential in vitro cardio- and hepato-toxicity resulting from drug (LB)–dietary supplement (FA/FT) association that might occur during pregnancy. This study was conducted using two immortalized healthy cell lines—H9c2(2-1) myoblasts and HepaRG hepatocytes. The selected cardiac cells have proliferated well in vitro and have been used as an in vitro model in cardiotoxicity analyses, studies regarding the metabolic activity of the heart and mechanisms involved in myocyte damage, as well as in evaluations of compound-induced toxic effects [26,27]. In particular, their applicability in various in vitro studies results from the close morphological resemblance of H9c2 cells to immature embryonic cardiomyocytes. The cardiotoxicity of a given drug can be assessed through the evaluation of cell parameters such as morphology and proliferation. According to a recent study by Witek et al., the response of H9c2 cells to cytotoxic agents is highly dependent on the number of cell passages, this in vitro model being reliable if only cultured at low passages [26]. Thereafter, the cells employed for this study were cultured at low passages to avoid any lack of reliability. HepaRG is an immortalized human hepatic progenitor cell line recently emerging as a useful in vitro model for toxicological evaluations, representing one of the most employed cell lines for the study of drug-induced toxicity [28,29,30]. Compared to primary hepatocytes, HepaRG cells are more advantageous in terms of availability and reproducibility [28].

Considering that pregnancy has been associated with profound physiological changes affecting drugs’ pharmacokinetics [31], the LB, FA, and FT concentrations (expressed as nM) used for the in vitro experiments were chosen based on previous studies illustrating their blood levels in pregnant women following oral administration [32,33]. Thereafter, the concentrations of interest for this study were as follows: LB—50 and 150 nM; FA—0.2 nM; FT—25 nM. Additionally, we tested high concentrations of FA and FT (50 nM).

First, the possible cytotoxicity caused by a prolonged treatment (72 h) of H9c2(2-1) and HepaRG cells with LB, FA, FT, and their combinations was evaluated. The results indicate a dose-dependent decrease in the percentages of viable H9c2(2-1) and HepaRG cells following their individual treatments with LB, FA, and FT (Figure 1). LB produced no significant alterations in terms of cell viability—the H9c2(2-1) and HepaRG percentages were up to 79% and 88%, respectively—while FA exerted a similar effect (cell viabilities of 82% and 81%, respectively) at the highest concentration tested (50 nM), which exceeds physiological FA blood levels [33]. FT, on the other hand, showed a stimulatory effect on the viability of H9c2(2-1) at 0.2 nM, which might be linked to the currently available reports indicating the benefits of FT supplementation during pregnancy in preventing congenital heart defects [34,35,36]. Furthermore, observational studies have suggested the ameliorative property of FT intake on cardiovascular morbidity and mortality [37].

The impact of the association of LB with FA on the viability of H9c2(2-1) and HepaRG cells was statistically non-significant (Figure 2A,B). Similarly, the viability of the HepaRG cells following the LB–FT treatments was comparable to the control (Figure 2B), suggesting good biocompatibility. Interestingly, the individual treatments with LB 50 nM and FT 25 nM led to viability values of 84% and 96% in the H9c2(2-1) cells, while their combination improved cell viability up to 127%. The in vivo administration of FA, which is known to be subsequently converted into active folate [9], has been found to mitigate the cardiotoxicity induced by several drugs, including celecoxib and doxorubicin [38,39]. Thereafter, our in vitro results suggest that FT treatment can alleviate the viability-reducing effects exerted by LB on healthy myoblasts. However, this protective property exerted by FT has been noticed only at low concentrations, while at high concentrations (LB 150 nM and FT 50 nM), their association led to cardiac cytotoxicity, with cell viability of ~68% (Figure 2A). Regarding cellular morphology analysis, in comparison to the control, no significant morphological changes were noticed at the end of the combined 72 h treatment, apart from a slight confluence reduction in the H9c2(2-1) cells (Figure 3) treated with LB 150 nM–FT 50 nM and in the HepaRG cells (Figure 4) treated with LB 150 nM–FA 50 nM, data that are in correlation with the viability results.

Further, the influence of LB, FA, FT, LB–FA, and LB–FT on wound regeneration was assessed using the H9c2(2-1) cell line. As can be seen in our results (Figure 5), the individual treatment of the H9c2(2-1) cells with LB (50 and 150 nM), FA (0.2 and 50 nM), and FT 25 nM inhibited wound healing following 24 h of treatment, while FT 50 nM slightly stimulated the motility of myoblasts. To the best of our knowledge, the effect of LB on wound healing has not been evaluated so far. However, other β-blockers were included in similar studies. One such example is the study conducted by Cuesta et al. showing that propranolol treatment delayed wound healing in vitro in primary human umbilical vein endothelial HUVEC cells [40]. Regarding the wound healing properties of FA, Pakdemirli et al. highlighted in a recent in vitro study on HUVEC cells that FA (at µM concentrations) is effective for improving endothelial damage [41]. According to our data showing a better healing property of FA at 50 nM compared to 0.2 nM, it can be assumed that high concentrations of FA are required to promote wound regeneration. The association between LB (50 and 150 nM) and FA 0.2 nM proved beneficial for the wound regeneration of myoblasts, a significant increase in cell migration being recorded at the end of the treatment (wound healing rates > 80%). The combined LB–FT treatment induced no significant impairment during the cardiac healing process in vitro.

## 5. Conclusions

The current study was proposed to evaluate the possible toxicity induced by labetalol association with folic acid and folate on cardiac and hepatic cells, considering their possible association in clinical practice. The main conclusion of the study is that in vitro LB–FA and LB–FT exerted no cardio- or hepato-toxicity at known plasmatic concentrations. On the contrary, LB–FT improved the viability of H9c2(2-1) myoblasts, while LB–FA demonstrated cardiac healing properties, suggesting a possible benefit for cardiac function resulting from their co-administration, which should be explored in future studies.

## Data Availability

The data presented in this study are available upon request from the corresponding author.
